# Rotationplasty with Vascular Reconstruction for Prosthetic Knee Joint Infection

**DOI:** 10.1155/2015/241405

**Published:** 2015-03-30

**Authors:** Masahide Fujiki, Shimpei Miyamoto, Fumihiko Nakatani, Akira Kawai, Minoru Sakuraba

**Affiliations:** ^1^Division of Plastic and Reconstructive Surgery, National Cancer Center Hospital East, Chiba 277-8577, Japan; ^2^Division of Plastic and Reconstructive Surgery, National Cancer Center Hospital, Tokyo 104-0045, Japan; ^3^Division of Musculoskeletal Surgery, National Cancer Center Hospital, Tokyo 104-0045, Japan

## Abstract

Rotationplasty is used most often as a function-preserving salvage procedure after resection of sarcomas of the lower extremity; however, it is also used after infection of prosthetic knee joints. Conventional vascular management during rotationplasty is to preserve and coil major vessels, but recently, transection and reanastomosis of the major vessels has been widely performed. However, there has been little discussion regarding the optimal vascular management of rotationplasty after infection of prosthetic knee joints because rotationplasty is rarely performed for this indication. We reviewed four patients who had undergone resection of osteosarcomas of the femur, placement of a prosthetic knee joint, and rotationplasty with vascular reconstruction from 2010 to 2013. The mean interval between prosthetic joint replacement and rotationplasty was 10.4 years and the mean interval between the diagnosis of prosthesis infection and rotationplasty was 7.9 years. Rotationplasty was successful in all patients; however, in one patient, arterial thrombosis developed and necessitated urgent surgical removal and arterial reconstruction. All patients were able to walk independently with a prosthetic limb after rehabilitation. Although there is no consensus regarding the most appropriate method of vascular management during rotationplasty for revision of infected prosthetic joints, vascular transection and reanastomosis is a useful option.

## 1. Introduction

Rotationplasty, in which the distal leg and foot are rotated axially 180 degrees and grafted to the femur to create a functional joint replacing the knee, has been frequently performed as an alternative to above-the-knee amputation for skeletally immature patients after resection of sarcomas of the lower extremity [1–7]. However, rotationplasty can also be performed as a function-preserving salvage procedure for adult patients with infected knee prostheses [8–11].

**Figure 1:**
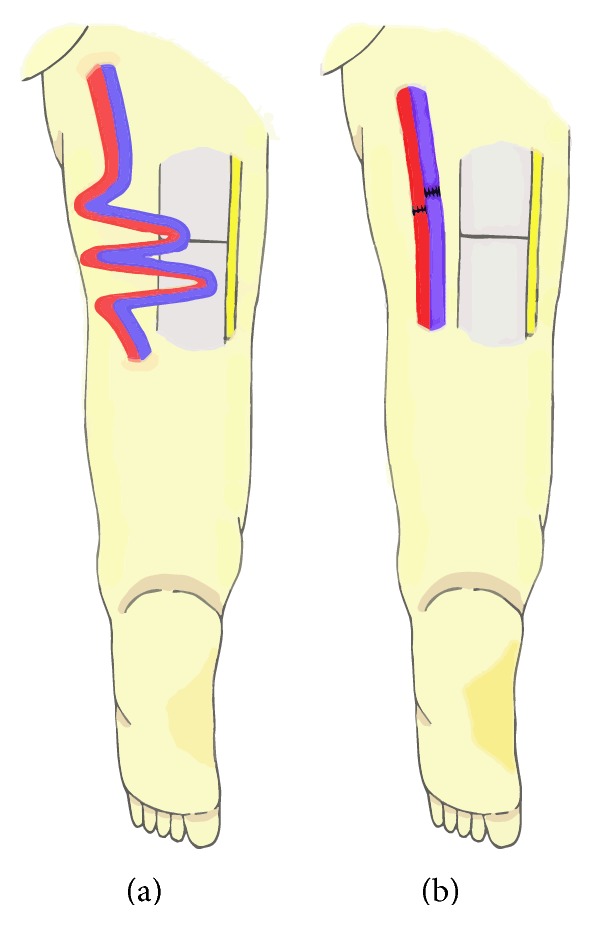
(a) Preservation and coiling of the major vessels, (b) transection and reanastomosis of the major vessels.

Conventional vascular management during rotationplasty is to preserve major vessels and coil them medially with the distal leg, but recently, the utility of transection and reanastomosis of the major vessels has been widely reported (Figure 1) [1, 2, 12–14]. However, no consensus has been reached regarding the optimal method of vascular management of rotationplasty after the resection of sarcomas [1, 12, 13]. Furthermore, there has been little discussion regarding the vascular management of rotationplasty for revision of infected prosthetic knee joints [1].

In the present study we reviewed four patients who had undergone rotationplasty with vascular reconstruction for revision of tumor prosthetic knee joints after long-term infections and summarize the relevant literature to guide management of such cases.

## 2. Patients and Methods

We reviewed four patients who underwent Van Nes rotationplasty, which allows continuation of longitudinal bone growth, for the revision of infected knee prostheses, at the National Cancer Center Hospital from 2010 to 2013. The patients were three men and one woman, with a mean age of 26.0 years (range, 17–45 years), who had received tumor prosthetic knee joint replacement after the resection of osteosarcomas of the femur. The mean interval between prosthetic joint replacement and rotationplasty was 10.4 years (range, 5.0–18.6 years) and the mean interval between the diagnosis of prosthesis infection and rotationplasty was 7.9 years (range, 2.7–18.3 years). All patients selected rotationplasty because of the pain and decreased range of motion of the infected prosthetic knee joints. The mean follow-up period after rotationplasty was 26.3 months (range, 10–44 months) and no patient was lost during follow-up.

The rotationplasty procedure was performed as follows. First, circumferential incisions were made in the skin of the distal thigh and proximal leg. Next, osteotomies were proximally and distally performed at the levels of the joint prostheses in the femur and the tibia, respectively. The sciatic nerve was then dissected and preserved along the entire length of the resection. Major vessels were excised en bloc with soft tissue around the knee joint, and the remnant leg and foot were externally and axially rotated 180 degrees. Femorotibial osteosynthesis was performed before vascular reconstruction and achieved via intramedullary nails or plate fixation. Vascular reconstruction was then performed under an operating microscope, and the order of arterial and venous anastomosis was determined in accordance with the intraoperative setting of vessels. Finally, the wound was closed in layers.

## 3. Results

The characteristics of the patients and operative details are summarized in Table 1. The mean operation time was 13.0 hours (range, 9.3–16.4 h). In three patients, the femoral artery and vein were anastomosed to the popliteal artery and vein, respectively. In one patient (patient 2), the femoral artery was anastomosed to the posterior tibial artery, and the femoral vein and its branch were anastomosed to the peroneal vein and the posterior tibial vein, respectively, because the vessels had been transected distally at the bifurcation of the popliteal vessels.

Although the postoperative course was uneventful in three patients, arterial compromise developed on the first postoperative day in one patient (patient 3). To restore blood flow, urgent surgical removal of a thrombus and arterial reconstruction with a vein graft were performed. On the other hand, no patients developed venous compromise after surgery.

Palsy of the sciatic nerve did not develop in any patient. In addition, all patients started rehabilitation approximately one week after rotationplasty; all were able to walk independently with a prosthetic limb during the last follow-up.

## 4. Representative Case 

Patient 2 was a 22-year-old man who presented with an infected prosthetic knee joint. When he was 11 years old, he underwent surgical excision with a wide margin and neoadjuvant chemotherapy for an osteosarcoma of the left femur and then received a prosthetic knee joint. However, an infection developed in the prosthetic joint 6 years later. Because of the pain and dysfunction of the affected limb, rotationplasty was performed at the patient's request.

During the operation, blood flow from the stump of the femoral artery was poor because of a thrombus of unknown cause in the proximal femoral artery; therefore, vascular reconstruction was performed after removal of the thrombus to resect the thrombosed femoral artery and perform end-to-end anastomosis.

**Figure 2:**
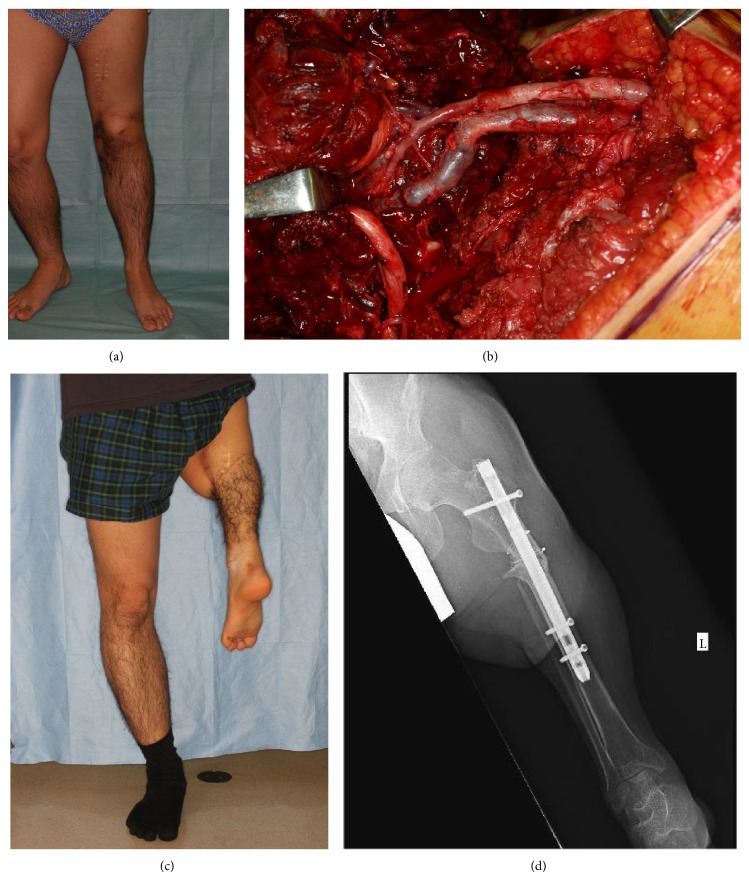
(a) Preoperative status with decreased range of motion due to infection of the prosthetic knee joint, (b) vascular reconstruction after rotation of the distal leg, (c) appearance 3-year after rotationplasty, (d) X-ray finding 3-year after rotationplasty.

The postoperative course was uneventful, and the patient started walking independently with a lower limb prosthesis 4 months after surgery. At 25 years of age, he is able to ride a bicycle and participate in sports while wearing the prosthesis (Figure 2).

## 5. Discussion

The most devastating complication after rotationplasty is vascular compromise of the rotated limb, which can result in eventual above-the-knee amputation or hip disarticulation. Although reported rates of vascular compromise after conventional rotationplasty range from 3.7% to 15.4% [1, 2, 12], the rate only after rotationplasty for revision of infected prosthesis has never been reported. In the conventional method of Van Nes rotationplasty, the femoropopliteal artery and vein are preserved with the sciatic nerve, the neurovascular structures are coiled, and the distal limb is rotated axially 180° and reattached to the proximal stump. However, these redundant and coiled vessels are susceptible to kinking and collapse, which sometimes result in vascular compromise of the distal limb. Therefore, some authors have suggested that, during rotationplasty, transection and reanastomosis of the vessels be performed [4, 14]. Although such a procedure requires technical expertise, loops of redundant vessels are removed, and the risk of kinking is reduced.

Regarding the optimal method of vascular management of rotationplasty after the resection of primary sarcomas, no consensus has been reached [12, 13]. However, vascular transection and reanastomosis would be the preferred vascular management method for rotationplasty performed for revision of infected prostheses. First, the presence of severe scar formation around the major vessels, caused by previous operations and long-term infection, could make dissection extremely difficult. En bloc resection that includes the major vessels may shorten the operation time and reduce the risk of inadvertent vessel injury. Second, the risk of vascular kinking is higher in cases of rotationplasty for revision of infected prostheses than in cases of rotationplasty for primary sarcomas when the vessels are preserved. Owing to scar formation, the major vessels lose elasticity and are more likely to become kinked [15].

The main disadvantage of transection and reanastomosis of the vessels is the risk of anastomotic failure. Although reported rates of vascular compromise after rotationplasty with vascular reconstruction range from 11.5% to 15.0% [2, 13, 16], the rate only after rotationplasty for revision of infected prosthesis has never been reported. In the present study, arterial thrombosis developed in one case; this is because of vascular kinking due to mismatch of the lengths between the remnant femur and the reconstructed vessels. In this case, we performed vascular anastomosis before osteosynthesis because the distal limb began to show signs of ischemia during resection. As a result, trimming of the artery was considered inappropriate and the artery became kinked. Therefore, we believe that osteosynthesis should be completed before vascular anastomosis unless acute revascularization is necessary.

We reviewed our four cases who had undergone rotationplasty with vascular reconstruction for revision of tumor prosthetic knee joints after long-term infections. Despite the small number of patients, the results of our cases are of interest because rotationplasty is rarely indicated for revision of infected prosthetic joints. Although there is little discussion regarding the most appropriate method of vascular management during rotationplasty for this indication, we consider that vascular transection and reanastomosis is better than vascular preservation.

## 6. Conclusions

Transection and reanastomosis of the major vessels is a useful option of vascular management during rotationplasty for long-term infection of knee prosthesis.

## Figures and Tables

**Table 1:** Patient characteristics and operative details.

Patient	Age (years), sex	Interval from joint replacement to rotationplasty (years)	Interval from infection to rotationplasty (years)	Operation time (hours)	Donor vessels	Recipient vessels	Perioperative complication	Follow-up after rotationplasty (months)
1	17, male	5.0	2.7	9.3	Femoral artery/vein	Popliteal artery/vein	None	44

2	22, male	12.0	6.0	13.2	Femoral artery	Posterior tibial artery	None	39
					Femoral vein	Peroneal vein		
					Branch of femoral vein	Posterior tibial vein		

3	45, female	18.6	18.3	16.4	Femoral artery/vein	Popliteal artery/vein	Arterial thrombosis	12

4	20, male	5.8	4.6	13.2	Femoral artery/vein	Popliteal artery/vein	None	10
